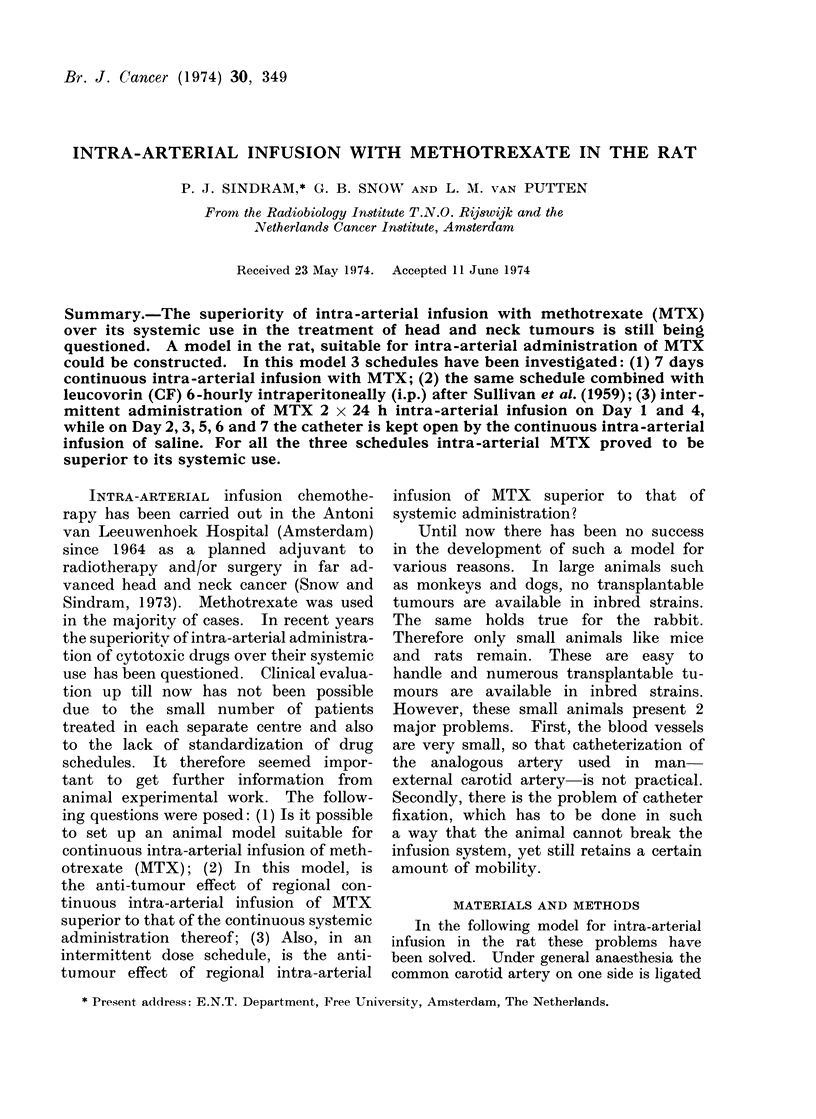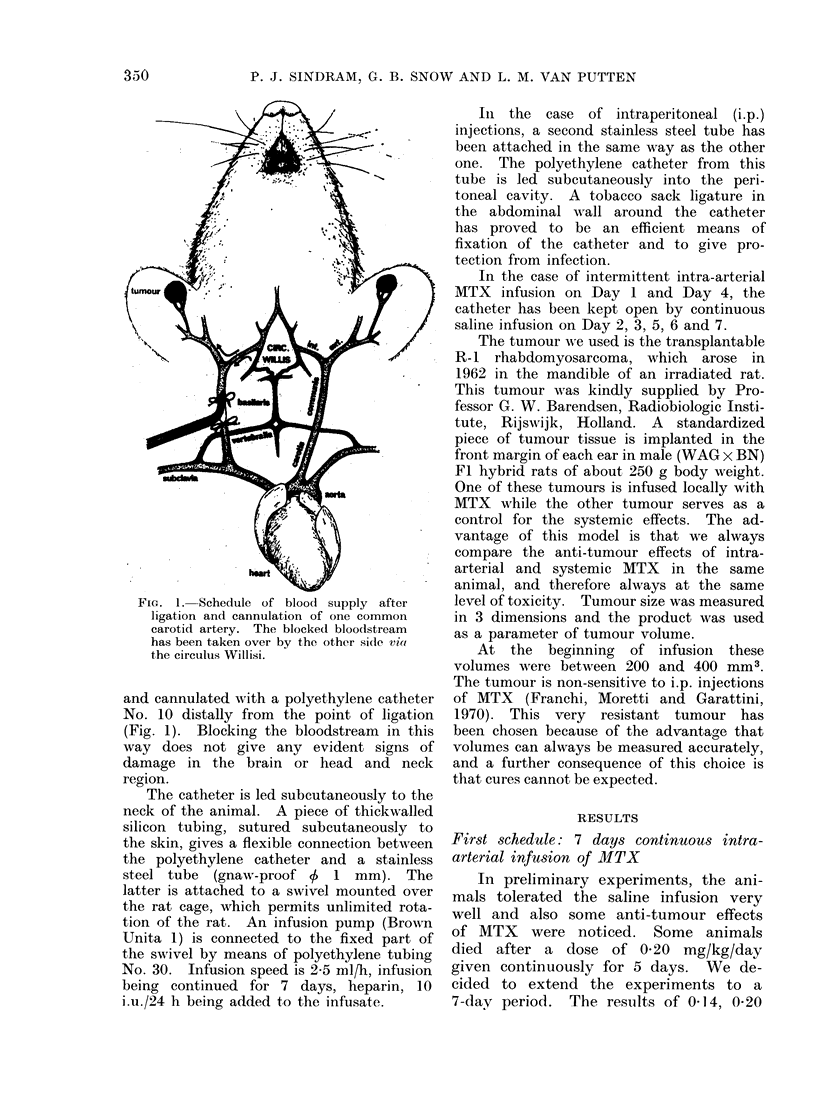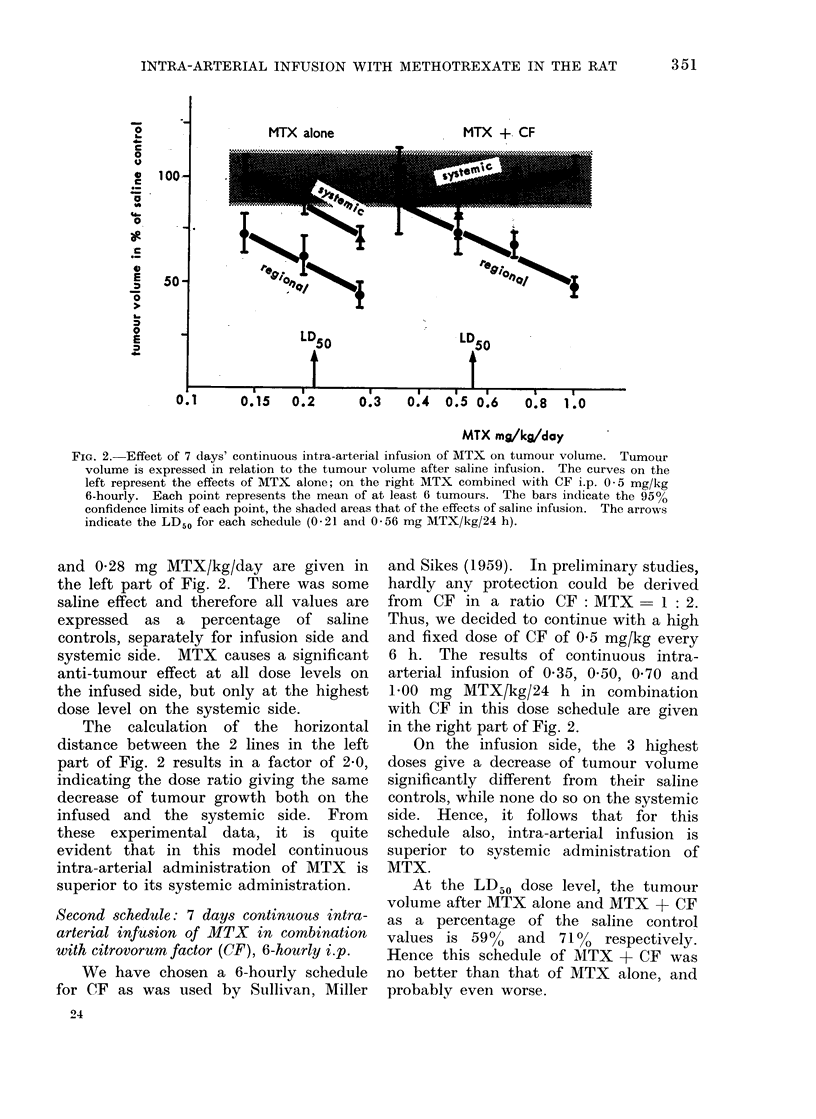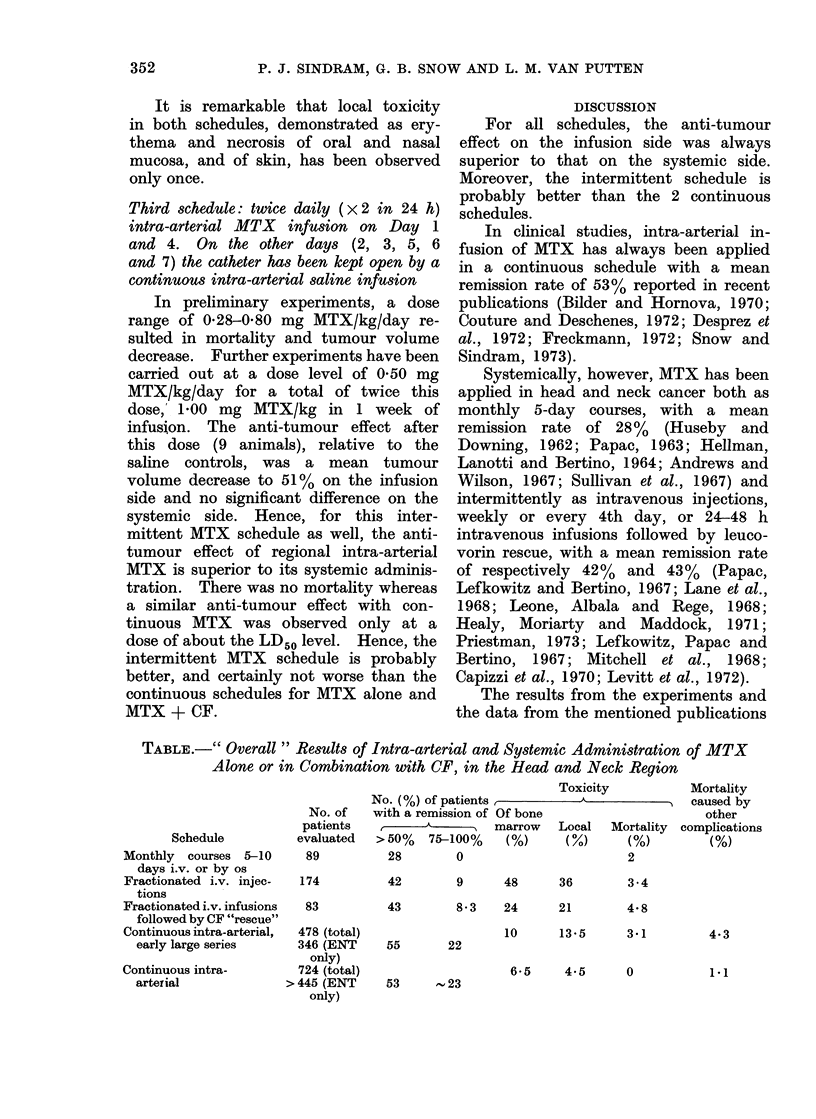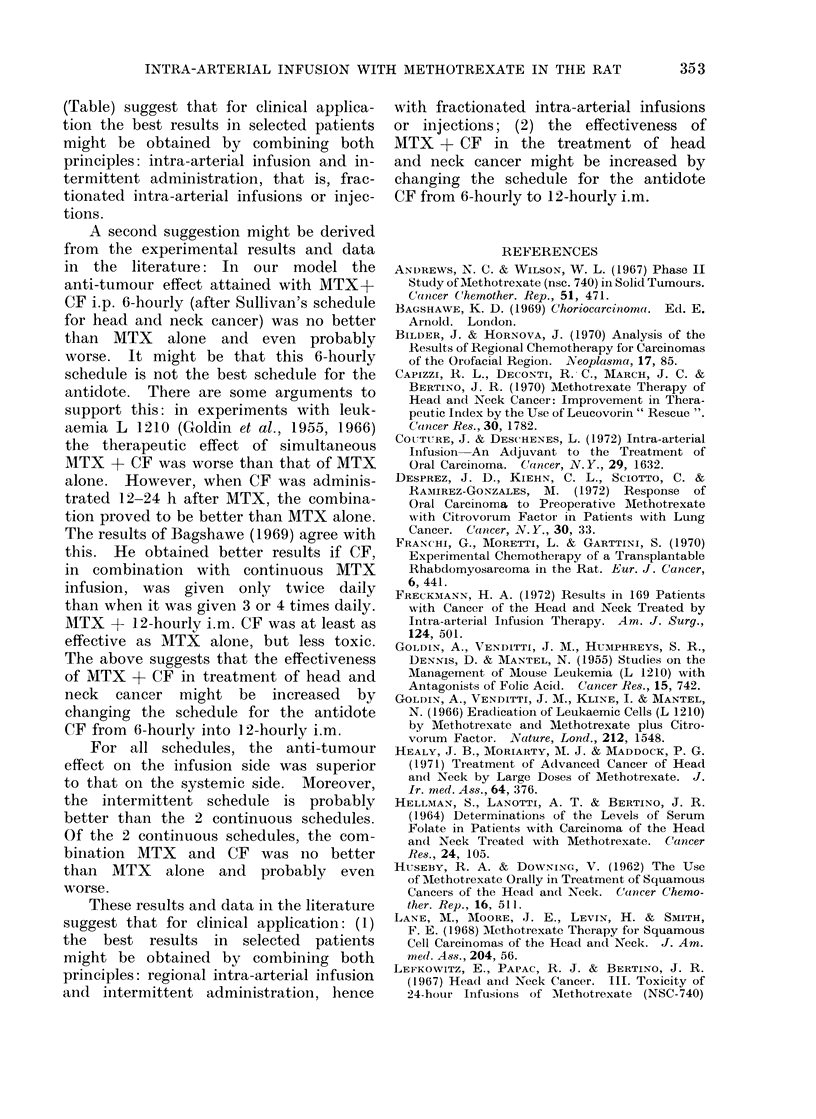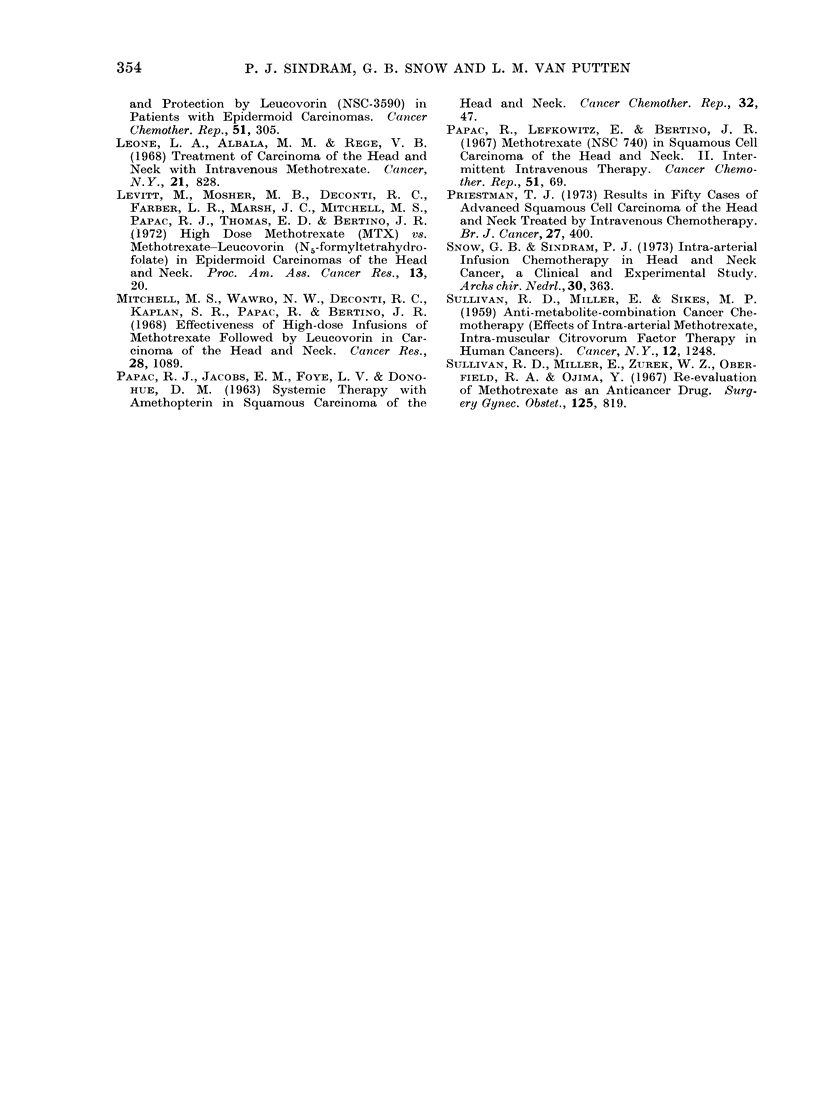# Intra-Arterial Infusion with Methotrexate in the Rat

**DOI:** 10.1038/bjc.1974.204

**Published:** 1974-10

**Authors:** P. J. Sindram, G. B. Snow, L. M. van Putten

## Abstract

The superiority of intra-arterial infusion with methotrexate (MTX) over its systemic use in the treatment of head and neck tumours is still being questioned. A model in the rat, suitable for intra-arterial administration of MTX could be constructed. In this model 3 schedules have been investigated: (1) 7 days continuous intra-arterial infusion with MTX; (2) the same schedule combined with leucovorin (CF) 6-hourly intraperitoneally (i.p.) after Sullivan *et al.* (1959); (3) intermittent administration of MTX 2 × 24 h intra-arterial infusion on Day 1 and 4, while on Day 2, 3, 5, 6 and 7 the catheter is kept open by the continuous intra-arterial infusion of saline. For all the three schedules intra-arterial MTX proved to be superior to its systemic use.


Br. J. Cancer (1974) 30, 349

### INTRA-ARTERIAL INFUSION WITH METHOTREXATE IN THE RAT

P. J. SINDRAM,* G. B. SNOW AND L. M. VAN PUTTEN

*From the Radiobiology Institute T.N.O. Rijswijk and the Netherlands Cancer Institute, Amsterdam*

Received 23 May 1974. Accepted 11 June 1974

**Summary.—The superiority of intra-arterial infusion with methotrexate (MTX) over its systemic use in the treatment of head and neck tumours is still being questioned. A model in the rat, suitable for intra-arterial administration of MTX could be constructed. In this model 3 schedules have been investigated: (1) 7 days continuous intra-arterial infusion with MTX; (2) the same schedule combined with leucovorin (CF) 6-hourly intraperitoneally (i.p.) after Sullivan *et al.* (1959); (3) intermittent administration of MTX 2 × 24 h intra-arterial infusion on Day 1 and 4, while on Day 2, 3, 5, 6 and 7 the catheter is kept open by the continuous intra-arterial infusion of saline. For all the three schedules intra-arterial MTX proved to be superior to its systemic use.**

INTRA-ARTERIAL infusion chemotherapy has been carried out in the Antoni van Leeuwenhoek Hospital (Amsterdam) since 1964 as a planned adjuvant to radiotherapy and/or surgery in far advanced head and neck cancer (Snow and Sindram, 1973). Methotrexate was used in the majority of cases. In recent years the superiority of intra-arterial administration of cytotoxic drugs over their systemic use has been questioned. Clinical evaluation up till now has not been possible due to the small number of patients treated in each separate centre and also to the lack of standardization of drug schedules. It therefore seemed important to get further information from animal experimental work. The following questions were posed: (1) Is it possible to set up an animal model suitable for continuous intra-arterial infusion of methotrexate (MTX); (2) In this model, is the anti-tumour effect of regional continuous intra-arterial infusion of MTX superior to that of the continuous systemic administration thereof; (3) Also, in an intermittent dose schedule, is the anti-tumour effect of regional intra-arterial infusion of MTX superior to that of systemic administration?

Until now there has been no success in the development of such a model for various reasons. In large animals such as monkeys and dogs, no transplantable tumours are available in inbred strains. The same holds true for the rabbit. Therefore only small animals like mice and rats remain. These are easy to handle and numerous transplantable tumours are available in inbred strains. However, these small animals present 2 major problems. First, the blood vessels are very small, so that catheterization of the analogous artery used in man—external carotid artery—is not practical. Secondly, there is the problem of catheter fixation, which has to be done in such a way that the animal cannot break the infusion system, yet still retains a certain amount of mobility.

### MATERIALS AND METHODS

In the following model for intra-arterial infusion in the rat these problems have been solved. Under general anaesthesia the common carotid artery on one side is ligated

* Present address: E.N.T. Department, Free University, Amsterdam, The Netherlands.

FIG. 1.—Schedule of blood supply after ligation and cannulation of one common carotid artery. The blocked bloodstream has been taken over by the other side *via* the circulus Willisi.

and cannulated with a polyethylene catheter No. 10 distally from the point of ligation (Fig. 1). Blocking the bloodstream in this way does not give any evident signs of damage in the brain or head and neck region.

The catheter is led subcutaneously to the neck of the animal. A piece of thickwalled silicon tubing, sutured subcutaneously to the skin, gives a flexible connection between the polyethylene catheter and a stainless steel tube (gnaw-proof $\phi$ 1 mm). The latter is attached to a swivel mounted over the rat cage, which permits unlimited rotation of the rat. An infusion pump (Brown Unita 1) is connected to the fixed part of the swivel by means of polyethylene tubing No. 30. Infusion speed is 2·5 ml/h, infusion being continued for 7 days, heparin, 10 i.u./24 h being added to the infusate.

In the case of intraperitoneal (i.p.) injections, a second stainless steel tube has been attached in the same way as the other one. The polyethylene catheter from this tube is led subcutaneously into the peritoneal cavity. A tobacco sack ligature in the abdominal wall around the catheter has proved to be an efficient means of fixation of the catheter and to give protection from infection.

In the case of intermittent intra-arterial MTX infusion on Day 1 and Day 4, the catheter has been kept open by continuous saline infusion on Day 2, 3, 5, 6 and 7.

The tumour we used is the transplantable R-1 rhabdomyosarcoma, which arose in 1962 in the mandible of an irradiated rat. This tumour was kindly supplied by Professor G. W. Barendsen, Radiobiologic Institute, Rijswijk, Holland. A standardized piece of tumour tissue is implanted in the front margin of each ear in male (WAG × BN) F1 hybrid rats of about 250 g body weight. One of these tumours is infused locally with MTX while the other tumour serves as a control for the systemic effects. The advantage of this model is that we always compare the anti-tumour effects of intra-arterial and systemic MTX in the same animal, and therefore always at the same level of toxicity. Tumour size was measured in 3 dimensions and the product was used as a parameter of tumour volume.

At the beginning of infusion these volumes were between 200 and 400 $mm^3$. The tumour is non-sensitive to i.p. injections of MTX (Franchi, Moretti and Garattini, 1970). This very resistant tumour has been chosen because of the advantage that volumes can always be measured accurately, and a further consequence of this choice is that cures cannot be expected.

### RESULTS

### *First schedule: 7 days continuous intra-arterial infusion of MTX*

In preliminary experiments, the animals tolerated the saline infusion very well and also some anti-tumour effects of MTX were noticed. Some animals died after a dose of 0·20 mg/kg/day given continuously for 5 days. We decided to extend the experiments to a 7-day period. The results of 0·14, 0·20

FIG. 2.—Effect of 7 days' continuous intra-arterial infusion of MTX on tumour volume. Tumour volume is expressed in relation to the tumour volume after saline infusion. The curves on the left represent the effects of MTX alone; on the right MTX combined with CF i.p. 0·5 mg/kg 6-hourly. Each point represents the mean of at least 6 tumours. The bars indicate the 95% confidence limits of each point, the shaded areas that of the effects of saline infusion. The arrows indicate the $LD_{50}$ for each schedule (0·21 and 0·56 mg MTX/kg/24 h).

and 0·28 mg MTX/kg/day are given in the left part of Fig. 2. There was some saline effect and therefore all values are expressed as a percentage of saline controls, separately for infusion side and systemic side. MTX causes a significant anti-tumour effect at all dose levels on the infused side, but only at the highest dose level on the systemic side.

The calculation of the horizontal distance between the 2 lines in the left part of Fig. 2 results in a factor of 2·0, indicating the dose ratio giving the same decrease of tumour growth both on the infused and the systemic side. From these experimental data, it is quite evident that in this model continuous intra-arterial administration of MTX is superior to its systemic administration.

*Second schedule: 7 days continuous intra-arterial infusion of MTX in combination with citrovorum factor (CF), 6-hourly i.p.*

We have chosen a 6-hourly schedule for CF as was used by Sullivan, Miller and Sikes (1959). In preliminary studies, hardly any protection could be derived from CF in a ratio CF : MTX = 1 : 2. Thus, we decided to continue with a high and fixed dose of CF of 0·5 mg/kg every 6 h. The results of continuous intra-arterial infusion of 0·35, 0·50, 0·70 and 1·00 mg MTX/kg/24 h in combination with CF in this dose schedule are given in the right part of Fig. 2.

On the infusion side, the 3 highest doses give a decrease of tumour volume significantly different from their saline controls, while none do so on the systemic side. Hence, it follows that for this schedule also, intra-arterial infusion is superior to systemic administration of MTX.

At the $LD_{50}$ dose level, the tumour volume after MTX alone and MTX + CF as a percentage of the saline control values is 59% and 71% respectively. Hence this schedule of MTX + CF was no better than that of MTX alone, and probably even worse.

It is remarkable that local toxicity in both schedules, demonstrated as erythema and necrosis of oral and nasal mucosa, and of skin, has been observed only once.

*Third schedule: twice daily* ($\times 2$ *in 24 h*) *intra-arterial MTX infusion on Day* 1 *and* 4. *On the other days* (2, 3, 5, 6 *and* 7) *the catheter has been kept open by a continuous intra-arterial saline infusion*

In preliminary experiments, a dose range of 0·28–0·80 mg MTX/kg/day resulted in mortality and tumour volume decrease. Further experiments have been carried out at a dose level of 0·50 mg MTX/kg/day for a total of twice this dose, 1·00 mg MTX/kg in 1 week of infusion. The anti-tumour effect after this dose (9 animals), relative to the saline controls, was a mean tumour volume decrease to 51% on the infusion side and no significant difference on the systemic side. Hence, for this intermittent MTX schedule as well, the anti-tumour effect of regional intra-arterial MTX is superior to its systemic administration. There was no mortality whereas a similar anti-tumour effect with continuous MTX was observed only at a dose of about the $LD_{50}$ level. Hence, the intermittent MTX schedule is probably better, and certainly not worse than the continuous schedules for MTX alone and MTX + CF.

### DISCUSSION

For all schedules, the anti-tumour effect on the infusion side was always superior to that on the systemic side. Moreover, the intermittent schedule is probably better than the 2 continuous schedules.

In clinical studies, intra-arterial infusion of MTX has always been applied in a continuous schedule with a mean remission rate of 53% reported in recent publications (Bilder and Hornova, 1970; Couture and Deschenes, 1972; Desprez *et al.*, 1972; Freckmann, 1972; Snow and Sindram, 1973).

Systemically, however, MTX has been applied in head and neck cancer both as monthly 5-day courses, with a mean remission rate of 28% (Huseby and Downing, 1962; Papac, 1963; Hellman, Lanotti and Bertino, 1964; Andrews and Wilson, 1967; Sullivan *et al.*, 1967) and intermittently as intravenous injections, weekly or every 4th day, or 24–48 h intravenous infusions followed by leucovorin rescue, with a mean remission rate of respectively 42% and 43% (Papac, Lefkowitz and Bertino, 1967; Lane *et al.*, 1968; Leone, Albala and Rege, 1968; Healy, Moriarty and Maddock, 1971; Priestman, 1973; Lefkowitz, Papac and Bertino, 1967; Mitchell *et al.*, 1968; Capizzi *et al.*, 1970; Levitt *et al.*, 1972).

The results from the experiments and the data from the mentioned publications

TABLE.—*" Overall " Results of Intra-arterial and Systemic Administration of MTX Alone or in Combination with CF, in the Head and Neck Region*

| Schedule | No. of patients evaluated | No. (%) of patients with a remission of >50% | No. (%) of patients with a remission of 75–100% | Toxicity: Of bone marrow (%) | Toxicity: Local (%) | Toxicity: Mortality (%) | Mortality caused by other complications (%) |
|---|---|---|---|---|---|---|---|
| Monthly courses 5–10 days i.v. or by os | 89 | 28 | 0 | | | 2 | |
| Fractionated i.v. injections | 174 | 42 | 9 | 48 | 36 | 3·4 | |
| Fractionated i.v. infusions followed by CF "rescue" | 83 | 43 | 8·3 | 24 | 21 | 4·8 | |
| Continuous intra-arterial, early large series | 478 (total) 346 (ENT only) | 55 | 22 | 10 | 13·5 | 3·1 | 4·3 |
| Continuous intra-arterial | 724 (total) >445 (ENT only) | 53 | ~23 | 6·5 | 4·5 | 0 | 1·1 |

(Table) suggest that for clinical application the best results in selected patients might be obtained by combining both principles: intra-arterial infusion and intermittent administration, that is, fractionated intra-arterial infusions or injections.

A second suggestion might be derived from the experimental results and data in the literature: In our model the anti-tumour effect attained with MTX+ CF i.p. 6-hourly (after Sullivan's schedule for head and neck cancer) was no better than MTX alone and even probably worse. It might be that this 6-hourly schedule is not the best schedule for the antidote. There are some arguments to support this: in experiments with leukaemia L 1210 (Goldin *et al.*, 1955, 1966) the therapeutic effect of simultaneous MTX + CF was worse than that of MTX alone. However, when CF was administrated 12–24 h after MTX, the combination proved to be better than MTX alone. The results of Bagshawe (1969) agree with this. He obtained better results if CF, in combination with continuous MTX infusion, was given only twice daily than when it was given 3 or 4 times daily. MTX + 12-hourly i.m. CF was at least as effective as MTX alone, but less toxic. The above suggests that the effectiveness of MTX + CF in treatment of head and neck cancer might be increased by changing the schedule for the antidote CF from 6-hourly into 12-hourly i.m.

For all schedules, the anti-tumour effect on the infusion side was superior to that on the systemic side. Moreover, the intermittent schedule is probably better than the 2 continuous schedules. Of the 2 continuous schedules, the combination MTX and CF was no better than MTX alone and probably even worse.

These results and data in the literature suggest that for clinical application: (1) the best results in selected patients might be obtained by combining both principles: regional intra-arterial infusion and intermittent administration, hence with fractionated intra-arterial infusions or injections; (2) the effectiveness of MTX + CF in the treatment of head and neck cancer might be increased by changing the schedule for the antidote CF from 6-hourly to 12-hourly i.m.

### REFERENCES

ANDREWS, N. C. & WILSON, W. L. (1967) Phase II Study of Methotrexate (nsc. 740) in Solid Tumours. *Cancer Chemother. Rep.*, **51**, 471.

BAGSHAWE, K. D. (1969) *Choriocarcinoma.* Ed. E. Arnold. London.

BILDER, J. & HORNOVA, J. (1970) Analysis of the Results of Regional Chemotherapy for Carcinomas of the Orofacial Region. *Neoplasma*, **17**, 85.

CAPIZZI, R. L., DECONTI, R. C., MARCH, J. C. & BERTINO, J. R. (1970) Methotrexate Therapy of Head and Neck Cancer: Improvement in Therapeutic Index by the Use of Leucovorin " Rescue ". *Cancer Res.*, **30**, 1782.

COUTURE, J. & DESCHENES, L. (1972) Intra-arterial Infusion—An Adjuvant to the Treatment of Oral Carcinoma. *Cancer, N.Y.*, **29**, 1632.

DESPREZ, J. D., KIEHN, C. L., SCIOTTO, C. & RAMIREZ-GONZALES, M. (1972) Response of Oral Carcinoma to Preoperative Methotrexate with Citrovorum Factor in Patients with Lung Cancer. *Cancer, N.Y.*, **30**, 33.

FRANCHI, G., MORETTI, L. & GARTTINI, S. (1970) Experimental Chemotherapy of a Transplantable Rhabdomyosarcoma in the Rat. *Eur. J. Cancer*, **6**, 441.

FRECKMANN, H. A. (1972) Results in 169 Patients with Cancer of the Head and Neck Treated by Intra-arterial Infusion Therapy. *Am. J. Surg.*, **124**, 501.

GOLDIN, A., VENDITTI, J. M., HUMPHREYS, S. R., DENNIS, D. & MANTEL, N. (1955) Studies on the Management of Mouse Leukemia (L 1210) with Antagonists of Folic Acid. *Cancer Res.*, **15**, 742.

GOLDIN, A., VENDITTI, J. M., KLINE, I. & MANTEL, N. (1966) Eradication of Leukaemic Cells (L 1210) by Methotrexate and Methotrexate plus Citrovorum Factor. *Nature, Lond.*, **212**, 1548.

HEALY, J. B., MORIARTY, M. J. & MADDOCK, P. G. (1971) Treatment of Advanced Cancer of Head and Neck by Large Doses of Methotrexate. *J. Ir. med. Ass.*, **64**, 376.

HELLMAN, S., LANOTTI, A. T. & BERTINO, J. R. (1964) Determinations of the Levels of Serum Folate in Patients with Carcinoma of the Head and Neck Treated with Methotrexate. *Cancer Res.*, **24**, 105.

HUSEBY, R. A. & DOWNING, V. (1962) The Use of Methotrexate Orally in Treatment of Squamous Cancers of the Head and Neck. *Cancer Chemother. Rep.*, **16**, 511.

LANE, M., MOORE, J. E., LEVIN, H. & SMITH, F. E. (1968) Methotrexate Therapy for Squamous Cell Carcinomas of the Head and Neck. *J. Am. med. Ass.*, **204**, 56.

LEFKOWITZ, E., PAPAC, R. J. & BERTINO, J. R. (1967) Head and Neck Cancer. III. Toxicity of 24-hour Infusions of Methotrexate (NSC-740)

and Protection by Leucovorin (NSC-3590) in Patients with Epidermoid Carcinomas. *Cancer Chemother. Rep.*, **51**, 305.

LEONE, L. A., ALBALA, M. M. & REGE, V. B. (1968) Treatment of Carcinoma of the Head and Neck with Intravenous Methotrexate. *Cancer, N.Y.*, **21**, 828.

LEVITT, M., MOSHER, M. B., DECONTI, R. C., FARBER, L. R., MARSH, J. C., MITCHELL, M. S., PAPAC, R. J., THOMAS, E. D. & BERTINO, J. R. (1972) High Dose Methotrexate (MTX) *vs.* Methotrexate–Leucovorin ($N_5$-formyltetrahydrofolate) in Epidermoid Carcinomas of the Head and Neck. *Proc. Am. Ass. Cancer Res.*, **13**, 20.

MITCHELL, M. S., WAWRO, N. W., DECONTI, R. C., KAPLAN, S. R., PAPAC, R. & BERTINO, J. R. (1968) Effectiveness of High-dose Infusions of Methotrexate Followed by Leucovorin in Carcinoma of the Head and Neck. *Cancer Res.*, **28**, 1089.

PAPAC, R. J., JACOBS, E. M., FOYE, L. V. & DONOHUE, D. M. (1963) Systemic Therapy with Amethopterin in Squamous Carcinoma of the Head and Neck. *Cancer Chemother. Rep.*, **32**, 47.

PAPAC, R., LEFKOWITZ, E. & BERTINO, J. R. (1967) Methotrexate (NSC 740) in Squamous Cell Carcinoma of the Head and Neck. II. Intermittent Intravenous Therapy. *Cancer Chemother. Rep.*, **51**, 69.

PRIESTMAN, T. J. (1973) Results in Fifty Cases of Advanced Squamous Cell Carcinoma of the Head and Neck Treated by Intravenous Chemotherapy. *Br. J. Cancer*, **27**, 400.

SNOW, G. B. & SINDRAM, P. J. (1973) Intra-arterial Infusion Chemotherapy in Head and Neck Cancer, a Clinical and Experimental Study. *Archs chir. Nedrl.*, **30**, 363.

SULLIVAN, R. D., MILLER, E. & SIKES, M. P. (1959) Anti-metabolite-combination Cancer Chemotherapy (Effects of Intra-arterial Methotrexate, Intra-muscular Citrovorum Factor Therapy in Human Cancers). *Cancer, N.Y.*, **12**, 1248.

SULLIVAN, R. D., MILLER, E., ZUREK, W. Z., OBERFIELD, R. A. & OJIMA, Y. (1967) Re-evaluation of Methotrexate as an Anticancer Drug. *Surgery Gynec. Obstet.*, **125**, 819.